# Adapting care provision in family practice during the COVID-19 pandemic: a qualitative study exploring the impact of primary care reforms in four Canadian regions

**DOI:** 10.1186/s12875-024-02356-x

**Published:** 2024-04-06

**Authors:** Maria Mathews, Lindsay Hedden, Julia Lukewich, Emily Gard Marshall, Leslie Meredith, Lauren Moritz, Dana Ryan, Sarah Spencer, Judith B. Brown, Paul S. Gill, Eric K. W. Wong

**Affiliations:** 1 Department of Family Medicine, Schulich School of Medicine & Dentistry, Western University, 1151 Richmond Street, London, ON N6A 5C1 Canada; 2 Faculty of Health Sciences, Simon Fraser University, 8888 University Drive, Burnaby, BC V5A 1S6 Canada; 3 Faculty of Nursing, Memorial University, 300 Prince Philip Drive, St. John’s, NL A1B 3V6 Canada; 4 Department of Family Medicine Primary Care Research Unit, Dalhousie University, 1465 Brenton Street, Suite 402, Halifax, NS B3J 3T4 Canada

**Keywords:** Primary care, Primary care reforms, Family physician, COVID-19, Pandemic response, Policy planning, Qualitative research

## Abstract

**Background:**

Over the past two decades, Canadian provinces and territories have introduced a series of primary care reforms in an attempt to improve access to and quality of primary care services, resulting in diverse organizational structures and practice models. We examine the impact of these reforms on family physicians’ (FPs) ability to adapt their roles during the COVID-19 pandemic, including the provision of routine primary care.

**Methods:**

As part of a larger case study, we conducted semi-structured qualitative interviews with FPs in four Canadian regions: British Columbia, Newfoundland and Labrador, Nova Scotia, and Ontario. During the interviews, participants were asked about their personal and practice characteristics, the pandemic-related roles they performed over different stages of the pandemic, the facilitators and barriers they experienced in performing these roles, and potential roles FPs could have filled. Interviews were transcribed and a thematic analysis approach was applied to identify recurring themes in the data.

**Results:**

Sixty-eight FPs completed an interview across the four regions. Participants described five areas of primary care reform that impacted their ability to operate and provide care during the pandemic: funding models, electronic medical records (EMRs), integration with regional entities, interdisciplinary teams, and practice size. FPs in alternate funding models experienced fewer financial constraints than those in fee-for-service practices. EMR access enhanced FPs’ ability to deliver virtual care, integration with regional entities improved access to personal protective equipment and technological support, and team-based models facilitated the implementation of infection prevention and control protocols. Lastly, larger group practices had capacity to ensure adequate staffing and cover additional costs, allowing FPs more time to devote to patient care.

**Conclusions:**

Recent primary care system reforms implemented in Canada enhanced FPs’ ability to adapt to the uncertain and evolving environment of providing primary care during the pandemic. Our study highlights the importance of ongoing primary care reforms to enhance pandemic preparedness and advocates for further expansion of these reforms.

**Supplementary Information:**

The online version contains supplementary material available at 10.1186/s12875-024-02356-x.

## Background

In Canada, individual provinces have responsibility for the organization and delivery of health care. Primary care is the first point-of-contact in the healthcare system and involves the delivery of comprehensive, accessible, longitudinal, and coordinated patient-centered care [1]. Primary care is largely delivered by family physicians (FPs) who are independent business owners or sub-contractors in the health system and who have traditionally been paid by fee-for-service (FFS) through province-run universal health insurance programs. Over the past two decades, provinces and territories have introduced a series of primary care reforms on an incremental, voluntary basis, resulting in a variety of organizational structures and practice models, with substantial variability across provincial jurisdictions [2–4]. Implemented reforms include changes to payment (or billing) models such as alternate models to FFS remuneration (collectively called alternative payment plans [APP]), formal patient enrolment or rostering, and performance incentives. In addition, there were changes to practice models such as the creation of larger group practices, interdisciplinary teams, linked and/or integrated practices, and expansion of primary care professionals, as well as the implementation of information technology including electronic medical records (EMRs) [2–9]. These reforms were framed as ways to improve access to and quality of primary care services.

Over the course of the COVID-19 pandemic, primary care practices in Canada had to adapt to a range of challenges that impacted their ability to safely deliver ongoing patient care. In March 2020, the volume of in-person primary care visits dropped as part of a larger closure of non-essential services amidst shortages of personal protective equipment (PPE) [10]. In addition, FPs had to manage patient care with restricted access to routine laboratory and diagnostic testing, specialist visits, or hospital-based services [11]. Out of necessity, FPs rapidly adopted virtual (primarily telephone) visits as public health insurance programs introduced or modified virtual care fee codes [10, 12]. Primary care practices implemented infection prevention and control (IPAC) procedures that allowed for the safe delivery of care by limiting the number of providers in office and altering patient flow to provide care when in-person was truly needed [13]. Over subsequent waves of COVID-19 infections (and closures of non-essential services), family practices provided a mix of virtual and in-person visits.

In this paper, we examine the impact of underlying primary care reforms on FPs’ ability to adapt to pandemic roles, including the provision of routine primary care. This study is part of a series of papers from a larger project exploring the pandemic from the perspective of FPs in Canada. The larger project is a multiple case study with cases consisting of: the Ontario (ON) Health West Region, the Vancouver Coastal health region in British Columbia (BC), the Eastern Health region in Newfoundland and Labrador (NL), and the province of Nova Scotia (NS). These regions, while pragmatically chosen as the locations of our pre-existing research team, vary in the organization and funding of primary care and reflect a variety of primary care reforms implemented across Canada [14].

## Methods

The protocol for the larger case study has been published elsewhere [14]. Using a case study approach [15], we conducted semi-structured qualitative interviews with FPs from October 2020 to June 2021. We invited FPs representing a wide range of personal characteristics (i.e., maximum variation sampling [16]) to participate in an interview until we had reached data saturation (i.e., sufficient data to allow for rigorous analysis and interpretation) [16, 17] based on post-interview debriefings and review of field notes during the data collection phase of the project. Throughout our recruitment, we considered characteristics such as having hospital and/or academic affiliations, genders, varied practice and funding models (e.g., FFS, APP), different community sizes, and different primary care practice settings (e.g., family practice clinics, long-term care facilities, and hospitals). To be eligible, FPs had to be licensed to practice independently at the time of the interview. We excluded FPs on temporary licenses or who held exclusively academic, research, or administrative roles. In each region, research assistants consulted faculty practice, team, and privileging lists, as well as the physician search portals of provincial medical regulators to identify potential participants. Research assistants posted recruitment notices in medical organizations’ newsletters, social media posts, and used snowball sampling (where permitted by local research ethics boards).

In each interview, we asked FPs to describe their personal and practice characteristics including their practice setting, primary funding model, and whether they work in an interdisciplinary team. With this context, we then asked them about the pandemic-related roles they performed over different stages of the pandemic and the facilitators and barriers to performing these roles. In the interviews, FPs organically discussed the impact of their organizational and funding models on their ability to adapt to the changing circumstances of providing patient care during the pandemic. Our questions and FP responses were based on the response to the pandemic at the time that we held the interviews (e.g., availability of vaccines, closure of schools, etc.). We carried out interviews using Zoom (Zoom Video Communications Inc.) or telephone as per the preference of the participant. A research assistant transcribed the recording of each interview.

Working independently, two or more members of the research team from each region read two to three transcripts (along with field notes from the interview) to identify key words and create codes, which were then organized into a preliminary coding scheme following discussion, in line with thematic analysis. The teams from each case compared the coding of four transcripts (one from each region) and, through discussion, developed a uniform coding template with code labels and descriptions that applied across all cases. We discussed disagreements in coding and coding descriptions until we reached an acceptable compromise. We used the unified coding template to code all transcripts and field notes, with the assistance of the NVivo 12 (QSR International) software. We used counts and proportions to describe participant characteristics. This paper presents findings from the codes related to primary care organization, funding, and integration with other health system organizations. We have also described the province and funding model (FFS, APP, or mixed) of each quoted participant.

In each region, research ethics boards approved the study. We obtained informed consent from participants before we scheduled and conducted interviews. We password protected files and securely stored recordings. We used participant identification numbers to help conceal the identity of individual participants.

### Positionality

We took a pragmatic approach in our research and made efforts to improve the quality of our data collection and analyses [16–18]. These efforts included pre-testing the interview questions with the FP members of our research team, documenting steps in the collection and analysis of the data, having experienced research assistants conduct interviews, and summarising responses back to participants to confirm meaning. We provided description of the context and meaning of quotes and looked for examples of data that presented opposing experiences or views. Our research team included FPs and public health officials, as well as researchers with extensive knowledge of primary care reforms in Canada, allowing us to draw on prior expert knowledge in the development of our interview guide, the development of the uniform coding template, and the interpretation of our results [15].

## Results

Among the 68 participants in the study, 41 (60.3%) were women, 49 (73.5%) had hospital privileges or affiliations, and 44 (64.7%) practiced in urban settings (Table 1).

**Table 1:** Characteristics of study participants

	Ontario	Nova Scotia	British Columbia	Newfoundland & Labrador	TOTAL
	*n* = 20	*n* = 21	*n* = 15	*n* = 12	*n* = 68
	n (%)	n (%)	n (%)	n (%)	n (%)
Gender^*^					
Men	10 (50)	9 (42.9)	4 (26.7)	4 (33.3)	27 (39.7)
Women	10 (50)	12 (57.1)	11 (73.3)	8 (66.7)	41 (60.3)
Funding Model					
Fee-for-Service	4 (20)	7 (33.3)	6 (40)	5 (41.7)	22 (32.4)
Alternative Payment Plan^**^	16 (80)	14 (66.7)	9 (60)	7 (58.3)	46 (67.6)
Hospital Affiliation					
No	5 (25)	6 (28.6)	3 (20)	5 (41.7)	19 (27.9)
Yes	15 (75)	15 (71.4)	12 (80)	7 (58.3)	49 (72.1)
Community Size^a^					
Rural	9 (45)	8 (38.1)	0 (0)	3 (25)	20 (29.4)
Small Urban	1 (5)	0 (0)	0 (0)	0 (0)	1 (1.5)
Urban	8 (40)	13 (61.9)	15 (100)	8 (66.7)	44 (64.7)
Mix	2 (10)	0 (0)	0 (0)	1 (8.3)	3 (4.4)
Years in Practice (mean)	18.7	15.4	16.9	16.3	16.9

^*^Gender was asked as an open-ended question

^**^Alternate payment includes all non-FFS or enhanced FFS funding models

^a^Rural ≤ 10,000 population, small urban = 10,000–99,999 population, Urban ≥ 100,0000 population

FPs identified reforms in five key areas that directly impacted their practices’ ability to operate and provide ongoing care during the pandemic: funding model, EMRs, integration with regional entities, interdisciplinary teams, and number of FPs in a group practice. All participants described their funding or practice model and referred to at least one of these key areas in their responses to interview questions.

Table 2 summarizes the state of reforms for each area of reform in each study site prior to the COVID-19 pandemic. As shown in Table 2, overall, FFS remained the predominant form of payment in BC and NL. Access to EMRs was high in all study provinces, except among FPs in NL. There was limited integration of FP practices with regional entities (such as a regional health authority, network, or local hospital), except in NS. With the exception of ON, team-based models of care were limited in BC, NS, and NL, where FPs predominately practiced in small groups of one or two physicians. In ON, team-based care was more widely integrated; teams were encouraged in Family Health Teams and reforms were conditional on forming co-located or virtual group practices of three or more physicians.

**Table 2:** Summary of selected primary care reforms in the four study regions

**British Columbia**	
Funding model	FFS funding accounted for 82% of payments to primary care physicians [19, 20]
EMR	By 2016, the majority of primary care practices used EMRs [8]
Integration with regional entities	FPs located within the same geographic region could be linked to a local Division of Family Practice that works with regional health authorities to provide services to meet the specific needs of their respective communities [21]
Interdisciplinary teams	Interdisciplinary team-based care was rare in community-based practice, despite being an ongoing focus of the “provincial vision” for primary care [22, 23]
Number of FP in a group	The majority of FPs in the province practiced in small group physician-owned and operated community practices that were “relatively isolated from other doctors and the larger healthcare system” [24]
**Ontario**	
Funding model	Reforms from the 2000s encouraged wider use of capitation with additional FFS and/or bonuses for targeted services for formally enrolled patients. Individual organizational models varied by the basket of services funded by capitation versus FFS payment [2, 5, 25]. By 2019, 44.8% of clinical services were paid by FFS [19]
EMR	Funding was available for FPs to implement EMRs [8]
Integration with regional entities	Local Health Integration Networks provided limited coordination and planning in the primary sector. In the fall of 2019, Ontario began the process of forming regional referral networks for hospital, medical, and community care services (called Ontario Health Teams), but had not fully implemented them when the COVID-19 pandemic was declared in March 2020
Interdisciplinary teams	Funding envelope for Family Health Teams included funding for other health professionals and administrative personnel [8]
Number of FP in a group	Funding reforms required forming groups with 3+ FPs [2]
**Nova Scotia**	
Funding model	In 2019, 41.3% of clinical payment in family medicine was billed through FFS [19]. Alternative payment consisted primarily of academic payment plans
EMR	The province incentivized EMR adoption using a fee code in the master billing agreement [3]
Integration with regional entities	In the capital region, NS created a District Department of Family Practice to strengthen primary care in the region
Interdisciplinary teams	Since the early 2000s, the province promoted nurse practitioner-led practices [26], collaborative FP and nurse practitioner, and FP registered nurse group practices [3]. The province introduced the Health Home Model in 2015 [27], consisting primarily of small teams of FPs, registered nurses, and/or nurse practitioners [3]
Number of FPs in a group	Primary care was largely delivered by FPs in solo or group practices, through collaborative family practice teams, or Community Health Teams [4]
**Newfoundland and Labrador**	
Funding model	The province relied heavily on FFS and salary for FPs who deliver the bulk of primary care services [19]. FFS accounted for 75.4% of all clinical payments to FPs [19]
EMR	Only 68% of physicians used an EMR [28]. An initiative to increase community-based physician access to the provincial EMR was introduced in 2016 [29]
Integration with regional entities	Salaried physicians were employees of regional health authorities. There was limited integration of FFS FPs prior to the pandemic
Interdisciplinary teams	Regional health authorities had not broadly integrated team-based models of care for salaried physicians [30]. In some rural/remote areas, community health centres offered more of an interdisciplinary presence, especially if virtual connections are considered [31]
Number of FPs in a group	No reforms related to FP group size

FFS – fee-for-service; EMR – electronic medical record; FP – family physician

### Funding model

Pandemic-related closures resulted in a sudden decrease to the volume of in-person patient visits and a rapid transition to virtual care. Participants noted that clinics funded by APP models were better able to financially navigate the sudden changes related to virtual care than FFS practices: “Patient volumes went down. Fortunately, because we’re capitation, the income stayed relatively the same. … if I was a fee-for-service physician, yeah, I would have ate my shirt” [ON10 APP]. The introduction of previously limited fee codes for virtual care were vital for FFS physicians: “having that ability to bill for a virtual visit, just from a practical financial standpoint, that was at least something, right? Because for a purely fee-for-service physician, if you didn’t have that, then you have zero, you have nothing” [ON16 FFS]. The introduction of the virtual fee codes had less impact on FPs who were funded through APPs: “we quickly adapted, as I mentioned because of our population-based funding model, an encounter is an encounter, it doesn’t matter if it’s by phone or a refill straight to the pharmacy or a discussion with a specialist colleague” [BC7 APP]. However, while provincial insurance plans quickly expanded the availability of virtual care billing codes, billing criteria in some provinces made it difficult for FFS FPs to generate sufficient revenue:

*We were going on the assumption that in each day we can get paid for a certain number of phone calls to patients to discuss more in-depth topics. But we get paid – we get $10 per phone call…You know, a regular in-office visit is $37. To go from getting paid a maximum capped per day of $10 per patient, … from a busy fee-for-service [practice], is pretty nerve-racking*. [NL7 FFS]

Moreover, participants noted that FFS remuneration for a virtual appointment with patients who had complex needs did not fully recognize the time required to provide care. There was considerable variation, even within the same region, in the adequacy of virtual care fees for complex care. While generally satisfied with the new fee codes, the participant explained:

*I was very pleased with how responsive the [provincial health insurance program] was in rolling out the new billing codes. …because my complex patients that take half an hour, I can bill a primary care HIV code, which is much higher paid than a primary care basic code.* [BC2 FFS]

However, the participant went on to state that slower changes to fee codes for addictions care resulted in uncompensated services: “We scaled-up pandemic withdrawal management, like the safe supply prescribing. … and there are no billing codes to match it, so we did it all for free” [BC2 FFS].

Participants also described the greater operating costs associated with providing care during the pandemic, which none of the funding models directly addressed. Of note, physicians paid through APP were better able to manage due to income stability. As summarized by a participant, “there needs to be clear provisions in a physician services agreement for pandemic care and for pandemic periods” [ON10 APP]. Additional costs stemmed from the IPAC requirements, such as the screening of patients: “I think the government needs to, or needed to, recognize that the costs of [providing care during a pandemic] are certainly much greater… when I look at the amount of time my staff spend on the phones screening patients” [ON13 APP]. They also needed to purchase PPE and cleaning supplies: “we had to purchase additional PPE, a whole bunch of different cleaning stuff and then we didn’t hire any additional staff, we just took on a lot of the tasks ourselves. … So, a lot more, certainly, work demands, which led to inefficiencies in seeing in-person people for sure” [ON10 APP]. Participants noted that they personally took on the additional cleaning requirements, which reduced their capacity to provide care because they could not afford to hire additional staff to perform these tasks: “at a time when we were earning less, we couldn’t afford to hire another person to come in just to do cleaning” [NL6 FFS].

### EMRs

The rapid shift to virtual care was facilitated by the existing use of EMRs: “so 70–80% of family docs now have access to the EMR” [NL8 APP] and “we already had remote access to our EMRs” [BC4 mixed]. However, participants noted that the move to virtual care required additional investment in infrastructure:

*There’s a cost involved with [virtual care] … to be able to provide virtual care with messaging and video and all that. There’s a supplemental cost. It works out to a few thousand dollars a year. I’m not sure as physicians whether we should say, ‘Well, that’s just the cost of business and we’re going to have to [absorb] that’ or whether there’s a role for the government…[NS5 APP]*.

Practices also incurred costs facilitating EMR access to all clinic staff who were working from home during the pandemic: “All my nurses, because they work from home, we have to set up an EMR for them at home. We have to make extra payment to the EMR people to give them access from home” [ON18 APP]. Without these additional changes to the EMR, staff would not have been able to provide virtual care during pandemic restrictions.

### Integration with regional entities

Participants described how integration (or, conversely, lack of integration) with a regional health authority, network, or local hospitals affected access to pandemic-related communication, PPE, and information and technology supports. For example, a participant whose primary care practice was one of the sites of the regional health authority commented that “we were in good communication with the [regional health authority] and they were very supportive” [NL3 APP]. In contrast, a participant without affiliation to the regional health authority recalled that for many months into the pandemic, “there was no formal communication between … the regional health authority and the community-based physicians” [NL11 FFS].

Participants who were integrated with the regional health authorities had better access to PPE and did not have to bear this additional cost (“I know there was a lot of discontent from other physicians about lack of PPE; I’m fortunate our clinic is funded by [the regional health authority], so we did have access to it … and it wasn’t an issue of having to pay for it” [NL4 APP]) unlike unaffiliated practices (“they didn’t provide personal protective equipment for us at all until this fall, maybe November, October, when they started giving it to us. So, we had nothing. And you couldn’t order it if you tried” [NS15 APP]).

Participants who belonged to a regional network also had access to computing support to help transition to online meetings (“[the network] rolled out like, an IT [information technology] support… It was like a virtual help desk …they have an IT guy specific for trying to get a Zoom up and running” [BC4 mixed]) and EMR upgrades to support virtual care:

*we were quite fortunate as a [regional health authority] site to, as opposed to an independent practice/fee-for-service practice … to have a relatively ready supply of PPE and then the [EMR], which we already had in place, and we were privy to the changes that they brought in* [NL2 APP].

In most provinces, COVID-related duties were first offered to providers who had pre-existing links with the regional entity that organized the assessment or vaccination clinics. In some provinces, performing pandemic-related duties was a condition of receiving income support and consequently, as one participant explained, FFS FPs in the region (who generally are not integrated with the regional health authority) were unlikely to benefit from the income supplement program:

*the income supplementation seemed to be helpful, but honestly, it was not good for most of us fee-for-service family physicians. We only got it if we did COVID work and this is where I feel that [our] regional health authority did not protect us very well … I know other [regional health authorities in the province] protected their family doctors by getting them to do COVID work …so that they would get the [income] supplementation*[NL6 FFS].

The participant noted that other regional health authorities in the province prioritized FFS physicians for COVID-related work to ensure that their incomes would be protected.

### Interdisciplinary team

Interdisciplinary practices were able to adapt more easily to IPAC guidelines, with specific team members taking the lead on updating and implementing processes for the practice: “it’s a collaborative clinic, which has an amazing family practice nurse, who’s well, well up on the latest [regional] policies and she’s very good to guide us through what’s required for personal protection equipment and sanitizing and those kinds of things” [NS11 APP]. In contrast, practices without teams had greater difficulty taking on these additional roles: “those practices where they really are fee-for-service, they don’t have the team, they don’t have the ability to respond in the way that we [a team-based practice] have” [ON3 APP].

While newer models of care include team-based care, non-physician health professionals are often employees of the local hospital or regional health authority assigned to work in primary care practices, rather than employees of the practice. As a result, a participant noted that individual practices had little control over the redeployment of members of their teams, leaving the practice short-staffed: “most of our nursing staff were redeployed and [our clinic] is really a nursing-run clinic, so that had a major impact” [BC5 APP].

### Number of FPs in a group practice

Participants also highlighted the benefits of belonging to a group practice with a larger number of FPs, which could afford to hire additional staff to take on some of the new roles required by IPAC protocols: “I’m in a large group, we have the availability of hiring on extra staff to screen everybody on the way in” [NS3 FFS]. Another participant noted that clinicians could devote more time to providing patient care while others took on leadership roles on behalf of the entire group:

*I felt lucky that we had a scale, enough of a size of our clinics that organizationally we had some people in place in leadership roles to take on doing that on our behalf … And the rest of us lowly clinicians could just keep going seeing patients while they were figuring that out for us. If you were a 1 or 2-person GP [general practitioner] practice with a nurse and an office in a building in wherever, you had nobody to do that, right?*[NS1 APP]

A participant in BC noted that the large number of FPs in their group practice meant they were better able to afford the extra costs associated with IPAC: “We’re a bit more fortunate because we are a group of nine practices now that collectively contribute to overhead. There was a pool of money that we were able to utilize” [BC7 APP].

## Discussion

Interviews with FPs during the COVID-19 pandemic suggest that practices that had adopted primary care reforms introduced in the past 20 years (namely APP models, EMRs, integration with regional entities, interdisciplinary teams, and increased number of FPs in a group practice; Table 2) were better able to implement pandemic-related adaptations in care. These reforms include both funding and practice model changes which are often linked (i.e., funding model changes that are tied to or facilitate changes in practice model). While none of the funding models fully covered the costs of providing care during the pandemic, belonging to an APP, large practice group, and/or an interdisciplinary team provided both financial and human resource capacity to absorb additional pandemic-related roles such as leadership, planning, and implementing IPAC protocols. Practices with prospective payment models (i.e., salaried, capitation, or blended capitation models) did not experience the same degree of cash flow restrictions as FFS practices. Across all case study regions, traditional solo or small group FFS practices had greater difficulty adapting the provision of routine primary care to the circumstances presented by the COVID-19 pandemic. Similar findings were reported among FFS primary care providers in other regions of Canada, the United States, and Australia [32–37]. An international study examining the use of virtual care in the first year of the pandemic found that the three countries (Canada [specifically the province of Ontario], Sweden, and the United Kingdom) with the highest rates of virtual primary care use in the pandemic period used capitation funding models; however, the study noted that funding model alone did not explain the higher utilization of virtual care [38]

Our findings highlight the value of greater integration of primary care practices with regional health entities [33]. Prior to the pandemic, practice networks in Ontario provided a critical mass to enable quality improvement, after-hours access, and economies of scales [5]. In addition to practical supports such as PPE, integration of family practices with regional entities facilitated planning and coordination and bi-directional communication between FPs and decision-makers in the four regions in our study [12, 39, 40], echoing findings from Alberta [33] and Australia [37].

The impact of the pandemic on primary care practices demonstrates the need for continued primary care reforms, including the expansion of alternate payment approaches, supports for virtual care, interdisciplinary teams, integration of primary care practices into regional entities, and greater numbers of FPs in a group practice. These reforms align with practice models favoured by FPs, especially recent graduates, [12, 32, 34, 41–43], are touted to reduce FP burnout [32, 44], and are reflected in new reforms in three of the four study regions. In 2023, BC announced increases in physician payment linked to time and patient complexity, and patient rostering [26], while NS’s recent reforms promote blended capitation payment, EMR use, minimum team size, and commitment to comprehensive primary care [26, 45]. The Health Accord NL [46] outlined efforts to integrate “collaborative care models” (now referred to as family care teams clinics) through interdisciplinary team-based care, rostering of patients to salaried physicians and/or nurse practitioners, and blended capitation funding models.

### Limitations

Our study is based on interviews conducted with the primary purpose of identifying FP pandemic roles and their supports and barriers. The interview guide did not explicitly ask about specific reforms in each region; nonetheless, the impact of primary healthcare reforms in each study site were readily apparent in the data as participants shared their stories. Moreover, unlike most FPs in Canada, the majority of FPs in the study were paid by APPs and had hospital affiliations; thus, our findings may under-represent the experiences of FFS and unaffiliated FPs. Primary care reforms also vary by province, so our findings may not represent the experiences of FPs outside our four case study provinces. Our study is based on self-reported data and may have been impacted by social desirability and recall bias [47, 48]. For example, participants may have been hesitant to discuss financial difficulties or appear to consider financial motives above community and patient needs.

## Conclusions

Primary care system reforms implemented in Canada over the last 20 years, namely APP models, EMRs, integration with regional health care entities, team-based models of care, and large group practices, enhanced FPs’ ability to adapt to the uncertain and evolving environment of providing primary care during the COVID-19 pandemic. Our study findings strengthen calls to expand these reforms within Canada.

### Electronic supplementary material

Below is the link to the electronic supplementary material.

Supplementary Material 1

## Data Availability

The datasets analysed for this study are not publicly available due to the need to maintain participant confidentiality; however, a portion of these data may be available from the corresponding author on reasonable request.

## Abbreviations

FP: Family physician
FFS: Fee-for-service
APP: Alternative payment plan
EMR: Electronic medical record
PPE: Personal protective equipment
IPAC: Infection prevention and control
BC: British Columbia
NL: Newfoundland and Labrador
NS: Nova Scotia
ON: Ontario
